# Biomechanical Analysis of Crossed Pinning Construct in Supracondylar Fracture of Humerus: Does the Point of Crossing Matter?

**DOI:** 10.7759/cureus.14043

**Published:** 2021-03-22

**Authors:** Ardilla Hanim, Muhammad Wafiuddin, Mohd Aizat Azfar, Mohd Shukrimi Awang, Nik Alyani Nik Abdul Adel

**Affiliations:** 1 Department of Orthopaedic, International Islamic University Malaysia, Kuantan, MYS; 2 Department of Orthopaedic, University Malaysia Sabah, Kota Kinabalu, MYS

**Keywords:** supracondylar humeral fracture, percutaneous pinning, biomechanical stability

## Abstract

Introduction

This appears to be the first biomechanical study that compares the stability of various locations of the crossing points in crossed pinning Kirschner wiring (K-wire) construct in treating pediatric supracondylar humerus fracture (SCHF). Additionally, this study compared the biomechanical stability between crossed pinning K-wire construct and the three-lateral divergent K-wire construct.

Methods

For the study purpose, 30 synthetic humerus bones were osteotomised at mid-olecranon fossa, anatomically reduced, and pinned using two 1.6-millimeter K-wires in five different constructs. A total of six samples were prepared for each construct and tested for extension, flexion, valgus, varus, internal rotation, and external rotation forces.

Results

As for crossed pinning K-wire construct, the center crossing point emerged as the stiffest construct in both linear and rotational forces, in comparison to the lateral crossing point, superior crossing, and medial crossing point

Conclusion

Based on this analysis, it is highly recommended that, if the crossed pinning construct is selected to treat supracondylar humerus fracture, the surgeon should aim for center crossing point as it is the most stable construct. Nevertheless, if lateral and superior crossing points are obtained during the initial attempt of fixation, the fixation may be accepted without revising the K-wire as the stability of these two constructs are comparable and portrayed no significant difference when compared to that of the center crossing point. Additionally, it is essential to avoid the medial crossing point as it is significantly less stable in terms of rotational force when compared to the center crossing point.

## Introduction

Supracondylar humerus fracture (SCHF) accounts for 18% of all pediatric fractures and it is the most common pediatric fracture around the elbow region. It commonly occurs in children aged between 5 and 10 years old. The increased involvement of girls in sports activities has led to gender predilection in terms of incidences of SCHF negligible and that SCHF mostly involves the non-dominant side [1-4]. The annual incidence of SCHF has been estimated to be 177.3 per 100,000 children [4].

The diaphysis of the humerus is connected to the epiphysis by the presence of a metaphysis flare. Although the medial and lateral columns for the distal humerus serve as strong pillars, they are connected by a thin zone of bone that is only 1 mm thick at the central portion. This central thin zone of the distal part of the humerus is formed by the olecranon fossa posteriorly and the coronoid fossa anteriorly. This distinct distal humeral anatomy is the reason that makes this area more vulnerable to sustain a fracture.

Supracondylar humerus fracture can be classified based on the direction of displacement of the proximal fragment. The extension type of SCHF, which happens when one falls on an outstretched hand and an extended elbow, may result in anterior displacement of a proximal fragment of the fracture. The flexion type of SCHF occurs from direct trauma to the posterior aspect of the distal humerus or from a fall on the olecranon with the elbow flexed and the proximal fragment displaced posteriorly. The extension type, which accounts for 97-99% of the cases, is far more common than the flexion type [1-4.]

Closed reduction and percutaneous fixation is the most common modality of treatment for SCHF and seems to be the treatment of choice for most Gartland Type II and III fractures; there are still certain areas of controversies or lack consensus [5]. Two widely accepted configurations for the pinning technique are the crossed pinning construct and the lateral divergent pinning construct. A substantial number of studies have compared the two constructs in terms of biomechanical stability, functional outcomes, and possible complications.

For crossed Kirschner wiring (K-wiring) configuration, a K-wire is inserted from the medial epicondyle and another K-wire is inserted from the lateral epicondyle. The crossing point of these wires must meet at the center of the humerus and located above the fracture region. The crossed pinning construct, despite carrying minimal risk of iatrogenic ulnar nerve injury, offers more stable fixation and is technically less demanding. Lateral divergent pinning construct was introduced mainly to minimize the risk of iatrogenic ulnar nerve injury. However, this construct is technically more demanding and was believed to have less biomechanical stability in the past. Nonetheless, both configurations have insignificant differences in terms of radiologic outcome, range of motion, and postoperative functional outcome [6]. In fact, there is no statistically significant difference between functional outcome and post-operative complications (ulnar nerve injury and loss of reduction) between lateral and crossed pinning constructs [7].

## Materials and methods

This biomechanical analysis was performed to test and compare the stiffness of different configurations of K-wire on artificial humeri bones. The methodology of this biomechanical analysis adhered to the standard procedures adapted from previous biomechanical analysis studies [8-12 ]. A total of 30 left synthetic humeri bones were used in this study. The synthetic bones were purchased from Synbone AG (Zizers, Switzerland).

**Figure 1:**
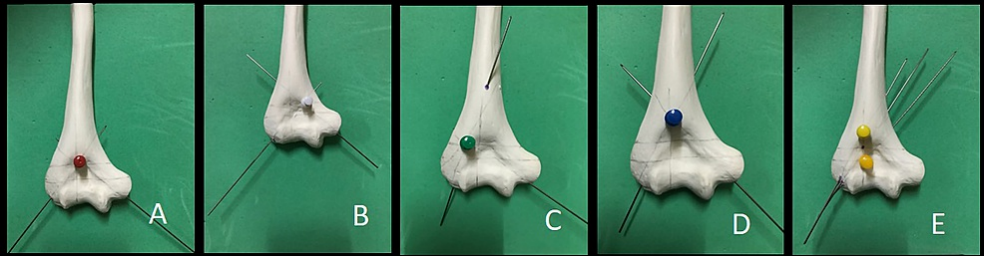
From left to right, centre crossing point (A), medial crossing point (B), lateral crossing point (C), superior crossing point (D), and lateral divergence (E)

Five K-wire configurations were tested. Four configurations were composed of center point crossing K-wire, medial point crossing K-wire, lateral point crossing K-wire, and superior point crossing K-wire, whereas another configuration was lateral divergent K-wire configuration (Figure 1).

Six samples were designated for each configuration and were tested for six different forces, namely, extension, flexion, valgus, varus, external rotation, and internal rotation forces. The mid-diaphyseal line was drawn first following the anatomical axis of the bone. This line was drawn by connecting at least three mid-diameter points along the diaphysis. A second line was drawn perpendicular to the mid-diaphyseal line at the center of the olecranon fossa measuring 2.5 cm from the most distal part of the humerus bone. This line denotes the osteotomy line. Next, the third and fourth lines were drawn 1 cm and 2 cm, respectively, proximal from the osteotomy line and perpendicular to the mid-diaphyseal line. All the constructs were made by predrilling into the synthetic bones with a 1.2 mm K-wire to ensure consistent pin placement between the specimens. These preliminary fixations were performed under image intensifier guidance to ascertain the consistency of the construct for all configuration types.

For all cross K-wire configurations, the starting point of the lateral pin was located at the center of the lateral epicondyle, while the starting point of the medial pin was situated at the inferior most aspect of the medial epicondyle. The center point refers to the point at the mid-diaphyseal line, which is 1 cm proximal to the osteotomy line. Both lateral and medial points were denoted by the point located 1 cm lateral and 1 cm medial, respectively, to the center point. Meanwhile, the superior point was the point located 1 cm superior to the center point at the mid-diaphyseal line. As for the lateral K-wire configuration, three K-wires were inserted with the starting point located at the capitulum. The first wire crossed the olecranon fossa at the mid-diaphyseal line and engaged the medial column. The second and third wires were directed more vertically to engage the lateral column and passed the mid-diaphyseal line 1 cm and 2 cm, respectively, proximal to the first wire. All preliminary fixations were removed, and all the samples were osteotomised by using a diamond tip saw following the osteotomy line. The final fixations were constructed by using K-wire at 1.6 mm following the predrilled holes.

The biomechanical testing was divided into two categories, namely, linear force testing and rotational force testing. Extension, flexion, valgus, and varus forces were categorized under linear force, whereas internal and external rotations were categorized under rotational force. For linear force mechanical testing, the sample was mounted to the universal tensile machine (UTM), Lloyd Instrument UK 10K+ machine (Ametek, Inc., Berwyn, PA, USA). The sample was mounted in such a way that the fulcrum of the machine was located 2 cm proximal to the osteotomy line and the load applicator was located at the distal fragment. Extension force testing was conducted by mounting the sample with its anterior surface facing upward, while flexion force testing was carried out by mounting the sample with its posterior surface facing upward. In valgus force testing, the sample was mounted with the medial surface facing upward and in varus force testing, the sample was mounted with the lateral surface facing upward. The load was applied to the distal fragment over the fulcrum at a displacement rate of 0.5 mm/s to a maximum of 4 mm. Both load (N) and displacement (mm) were measured at the distal fragment and recorded.

Each designated sample was tested five times, for a total of 100 linear force mechanical tests. The rotational force was tested for both internal and external rotations with the distal fragment clamped with two flat plates and connected to the machine. A special jig was designed and used to hold the proximal part of the sample. Manual torsion was applied at an angular displacement rate of 0.5 degree/s to an endpoint of ±10 degrees using WP 500 Torsion Testing Apparatus (GUNT Hamburg, G.U.N.T. Gerätebau GmbH, Barsbuttel, Germany). Both torque (Newton meter) and angular rotation (millimeter) were recorded. Each designated sample was tested for five times, for a total of 50 rotational force mechanical testing.

## Results

The analyses were performed in RStudio 1.0.153 (R Foundation, Vienna, Austria) using the Stats Package for Wilcoxon signed-rank test and ggpubr package for boxplots. The mean stiffness was used to describe the stiffness of each construct. The stiffness for each construct was compared with the center crossing point pinning construct. The constructs were then categorized into two types of force, namely linear and rotational forces, and were compared accordingly. Statistically, the median was used to compare lateral crossing point, medial crossing point, superior crossing point, and lateral divergence construct with center crossing point as the data were not normally distributed. Wilcoxon signed-rank test was employed for the second objective, which compared the stiffness of construct based on individual and categorical forces. The alpha value of 0.05 was set as the significance level.

Table 1 tabulates the mean stiffness for each construct for flexion, extension, valgus, varus external, internal, and linear forces. For flexion forces, the center crossing point emerged as the stiffest construct (36.749 N/mm) while the lateral divergence construct was the least stiff construct (26.914 N/mm) in resisting flexion force. Statistically, when compared with the center crossing point construct as the baseline, a significant difference was noted for medial crossing point (p=0.07937), superior crossing point (p=0.007927), and lateral divergence construct (p=0.007927). However, no significant instability difference was observed for the lateral crossing point construct (p=0.9999).

**Table 1:** Data comparing mean stiffness (N/mm) in different type of forces for each type of construct

Type of construct/Mean Stiffness (N/mm)	Flexion	Extension	Valgus	Varus	External Rotation	Internal Rotation	Linear	Rotational
Centre	36.749	51.485	35.550	71.000	0.380	0.390	48.6960	0.385
Lateral	36.714	52.838	32.522	83.384	0.410	0.360	47.6235	0.380
Medial	32.809	50.908	29.504	67.860	0.410	0.350	43.5952	0.380
Superior	30.743	40.432	24.378	66.422	0.400	0.350	41.3335	0.380
Divergence	26.914	27.573	23.393	58.684	0.230	0.300	38.7175	0.265

For extension forces, the superior crossing point appeared to be the stiffest construct (52.838 N/mm), whereas the lateral divergence construct was the least stiff construct (27.573 N/mm) in resisting extension force. Statistically, with the center crossing point construct serving as the baseline, significant instability difference was observed for lateral crossing point (p=0.007937) and lateral divergence (p=0.007937). However, an insignificant instability difference was found for the superior crossing point (p=0.1425) and medial crossing point (p=0.5476). 

For valgus forces, the center crossing point was the stiffest construct (35.550 N/mm), while the medial crossing point construct was the least stiff construct (23.393 N/mm). A significant instability difference derived statistically was noted between the center crossing point and all the other pinning constructs (p<0.05). 

For varus forces, the medial crossing point was the stiffest construct (83.384 N/mm) and appeared to be significantly more stable (p=0.007937) than the center crossing point statistically. The lateral crossing point was the least stiff construct (58.684 N/mm). Significant instability difference was found in lateral divergence construct (p=0.007937), superior crossing point (p=0.007937), and divergent point construct (p=0.03175) when compared with the center crossing point construct

The stiffest constructs for external rotation forces were the lateral divergent construct and the lateral crossing point, as they displayed similar mean stiffness (0.410 Nmm/degree). The medial crossing point construct, on the other hand, was the least stiff construct (0.230 Nmm/degree) in resisting external rotation force. Statistically, significant instability difference was found in medial crossing point construct (p=0.008562), when compared to center crossing point construct. The other constructs, however, exhibited an insignificant difference in terms of stability in resisting external rotation force, when compared to center crossing point (p>0.05)

For internal rotation force, the stiffest and the least stiff constructs were the center crossing point (0.390 Nmm/degree) and the medial crossing point (0.300 Nmm/degree), respectively, in resisting internal rotation force. Statistically, significant instability difference was observed for lateral crossing point (p=0.08326), medial crossing point (p=0.01175), and divergence construct (p=0.01996) when compared to center crossing point construct as the baseline. The superior crossing point gave no significant difference (p=0.09296) in terms of stability in resisting internal rotation force when compared to the center crossing point

For linear force, the centre crossing point construct was the stiffest construct (48.6960 N/mm), whereas the divergence construct was the least stiff construct (38.7175 N/mm) in resisting linear force. Significant instability difference was noted for lateral divergence construct (p=0.0007474) when compared to the centre crossing point construct statistically. Nevertheless, medial, superior, and lateral crossing points showed no significant difference (p>0.05) in terms of stability in resisting linear force, when compared to the centre crossing point

For rotational forces, the mean stiffness shows that the centre crossing point construct was the stiffest construct (0.385 Nmm/degree), while the medial construct was the least stiff construct (0.265 Nmm/degree) in resisting rotational force. Statistically, with the centre crossing point construct selected as the baseline, a significant instability difference was found for the medial crossing point construct (p=0.0001351). Meanwhile, lateral crossing point, superior crossing point, and divergence constructs exhibited insignificant difference (p>0.05).

## Discussion

Closed manipulative reduction and percutaneous pinning is the gold standard treatment for displaced SCHF in children, and it is one of the most widely applied procedures in pediatric orthopedics [5]. The two commonly accepted configurations for the pinning technique are the crossed pinning construct and the lateral divergent pinning construct.

Lateral divergent pinning construct is typically used to minimize the risk of iatrogenic ulnar nerve injury and seems to be the preferred technique, especially by the pediatric orthopedic fraternity. Most of the recent studies have recommended lateral pinning construct over cross pinning construct in view of the risk of iatrogenic ulna nerve injury [5]. Lateral pin fixation offers not only similar functional and radiological outcomes but also almost equal mechanical stability when compared with medial-lateral pinning without the risk of iatrogenic ulnar nerve injury [7]. Many biomechanical analyses have compared the stability of various constructs of lateral pinning. A biomechanical analysis found that divergent lateral K-wire is more stable than parallel lateral wire [8].

Centre crossing point in supracondylar humerus pinning must be 1.5-2.0 cm above the fracture line and both wires purchase the opposite cortex [7]. Nevertheless, the technique does not mention exactly where the crossing point should be placed to attain stability. In order to get the center crossing point, attending surgeons, at times, face difficulty in getting the perfect center crossing point of the medial and lateral K-wires, which subsequently leads to multiple attempts during K-wires insertion. Multiple attempts of pinning are expected during the procedure as one tries to get a centrally located crossing point construct. Multiple attempts, nonetheless, may increase the risk of ulnar nerve injury. Besides, such attempts can weaken the pin bone interphase and the hold of K wire [5], apart from increasing the exposure of direct and scattered radiation from the c-arm during the procedure [13].

In fact, many studies have concluded that the occurrence of iatrogenic ulnar nerve injuries following crossed pinning technique ranges from 1.1% from one study to 9.0%, in which all patients recovered within six weeks with physiotherapy [14]. Crossed pin fixation using a mini-open approach is an appropriate and safe treatment option for displaced SCHF, as it does not add additional morbidity to the procedure [15].

Eight SCHFs with loss of reduction were analyzed to identify the causative factors [16]. In all the cases, loss of reduction had been due to identifiable errors on the intraoperative fluoroscopic images that were classified into three types: (1) failure to engage both fragments with two pins or more, (2) failure to achieve bicortical fixation with two pins or more, and (3) failure to achieve adequate pin separation (>2 mm) at the fracture site. A study showed that in the hands of junior trainees in their first three years of training, more stable fixation was achieved with cross pinning when compared with the lateral pinning technique [17].

Besides, there has been an ongoing debate on the choice of pin configuration while fixing SCHF. In laboratory settings, cross pinning projects better stability, but in a clinical setting, both seem to do equally well [8]. In fact, there is an insignificant difference in outcomes for loss of carrying angle and range of motion between the mediolateral pinning group and lateral pinning group at the end of six months [17]. Both configurations displayed no significant difference in terms of radiologic outcome, range of motion, and post-operative [6,7]. Hence, this present study identified and compared the biomechanical stability in various constructs of crossed pinning techniques, in order to provide treating surgeons information on the most optimal crossing point construct for SCHF in children.

Four types of linear forces (flexion, extension, valgus, and varus) and two types of rotational forces (internal rotation and external rotation) were tested for each configuration in this study. When comparing the different locations of the crossing point in crossed pinning construct, the center crossing point emerged as the stiffest construct in both linear and rotational forces (48.6960 N/mm; 0.385 Nmm/degree), when compared with lateral crossing point (41.335 N/mm; 0.380 Nmm/degree), superior crossing point (43.5952; 0.380 Nmm/degree), and medial crossing point (47.6235 N/mm; 0.265 Nmm/degree). This is true as the center crossing point displayed an almost equal moment arm from medial and lateral sides, which distributed the load more effectively in resisting both rotational and linear forces. In view of a specific type of force, the center crossing point was not always the stiffest construct. In extension and varus forces, superior and medial crossing points were the most stable constructs, respectively. In fact, the center crossing point was ranked at the third position in external rotation force. This is attributed to the distal humerus anatomy, which is not in a perfect triangular shape to resist different forces in an equal manner. The medial supracondylar pillar subtends an angle of approximately 20° with a long axis of the shaft, while the lateral pillar subtends an angle of 35-40° with the shaft axis. This explains why distal humerus reacted differently for different types of force. However, on average, the center crossing point is overall the stiffest construct in both linear and rotational forces.

Although the center crossing point has been verified as the stiffest construct, lateral and superior crossing points displayed insignificant statistical differences when compared with the center crossing point for both linear and rotational forces. On the other hand, the medial crossing point showed no statistically significant difference for linear force, but it demonstrated a statistically significant difference for rotational force when compared with the center crossing point. To the best of the authors’ knowledge, no study has assessed the various locations of the crossing points to determine the stability of the crossed pinning construct. Many recent biomechanical studies seem to focus more on the difference between crossed pinning technique and lateral pinning technique or on the various configurations in the lateral pinning technique.

Apart from comparing the stiffness between various locations of crossing points for crossed pinning construct, a stiffness comparison was also made in this study between the center crossing point construct and the lateral divergent construct. Previous biomechanical studies support the concept that a medial pin is required for better fixation. A cadaveric study had demonstrated that medial and lateral crossed pins were superior to lateral pin configurations, including both two and three lateral pins [12]. Similarly, the outcomes from this present study indicated that the center crossing point construct was superior to the lateral divergent construct for both linear and rotational forces.

This present analysis has generated comparable and consistent results with other recent biomechanical studies. Further prospective clinical studies to compare among various crossing point locations in terms of functional outcome, risk of fixation failure, and possible complications could be initiated.

It is noteworthy to highlight two limitations in this study. Despite the use of the largest number of specimens compared to the other biomechanical studies, the sample size is still considered small. In this study, each specimen was tested for a single type of force, but the test was repeated five times to obtain the average value. Ideally, for a more accurate result, five different specimens for each construct should be tested for a single type of force. The other limitation of this study refers to the use of synthetic humeri bone instead of cadaveric bone. Although the absolute values for linear and torsional stability of synthetic humeri differ from the pediatric humeri in vivo, synthetic humeri do provide anatomically accurate mean values in determining the relative linear torsional stability provided by the various pin configurations.

## Conclusions

This study validates that, in the group of crossed pinning constructs, the center crossing point emerged as the stiffest amongst other crossing points. Nevertheless, the stability displayed by superior and lateral crossing points appeared to be comparable with that of the center crossing point. This study has proven that the stability of the lateral divergent construct is indeed comparable to that of crossed pinning construct.

From this analysis, it is highly suggested that, if the crossed pinning construct is selected as the treatment option for SCHF, the surgeon should aim for the center crossing point as it is the most stable construct. However, if lateral and superior crossing points are obtained during the initial attempt of fixation, they may be accepted without revising the K-wire, mainly because the stability of these constructs was comparable and exhibited no significant difference when compared with the center crossing point. Another recommendation that can be derived from this study is that it is best to avoid the medial crossing point as it is significantly less stable in terms of rotational force when compared to the center crossing point.
